# The Signal in the Genomes

**DOI:** 10.1371/journal.pcbi.0020035

**Published:** 2006-04-28

**Authors:** David Sankoff

Nostra culpa. Not only did we foist a hastily conceived and incorrectly executed simulation on an overworked RECOMB conference program committee, but worse—*nostra maxima culpa*—we obliged a team of high-powered researchers to clean up after us! It was never our intention to introduce an alternative way of constructing synteny blocks; the so-called ST-synteny was only a (bungled) attempt to mimic Pevzner and Tesler's method, based on our reading or misreading of their paper [1]. Moreover, shortly after the conference, before preparing the full journal version of our article, we recognized through a back-of-an-envelope calculation that realistic values of the parameters in our simulations would not produce much increase in reuse rate. Consequently, our published article [2] develops only the main part of our communication, modeling and simulating the artifactual increase in reuse rates due to deleting synteny blocks but not that due to the construction of synteny blocks.

Unfortunately, our makeshift work distracted from the main point of our communication. The theme in our full article [2], in the RECOMB extended abstract, and elsewhere is not substantially confronted in the recently published *PLoS Computational Biology* paper by Glenn Tesler and colleagues [3]. Wherever high rates of breakpoint reuse are inferred, whether they are due to bona fide reuse or rather to violations in the assumptions justifying the use of particular algorithms (relating to the construction of synteny blocks or their size thresholds, or to the unrealistically limited repertoire of rearrangement processes recognized by the algorithm), there is a correspondingly high rate of loss in the historical signal.

While two genomes diverge without breakpoint reuse, the historical signal is conserved in the breakpoint graph, which consists entirely of four-vertex cycles, specifying exactly which pairs of breakpoints must be healed by reversals or translocations. As breakpoints are reused—as they eventually must be for finite gene orders, or for genomic sequence, where there are criteria for deciding when two breakpoints are too close together to be considered distinct—the four-vertex cycles are merged into larger structures, and the breakpoint graph becomes ambiguous concerning the rearrangements that produced it. The two divergent genomes eventually become randomized with respect to each other. But this randomization also occurs, even if divergence involves only distinct breakpoints, when the assumptions underlying the use of genome rearrangement algorithms are violated, which can happen in many possible ways [4,5]. And we cannot infer whether mutually randomized synteny block orderings derived from two divergent genomes were created through bona fide breakpoint reuse or rather through noise introduced in block construction or through processes other than reversal and translocation.

I illustrate this point with data on the human/mouse comparison from Pevzner and Tesler's more detailed paper [6]. We simulated 100 pairs of genomes constructed of 22 and 19 human and mouse autosomes, with 270 blocks distributed exactly as in the human and mouse genomes, except that the blocks were randomly permuted and sign—or strandedness—was assigned randomly to each block. Permutations are within, not between, chromosomes, assuring a realistic reversals/translocations ratio. Output from the standard rearrangement algorithm [7] is summarized in Table 1.

**Table 1:**
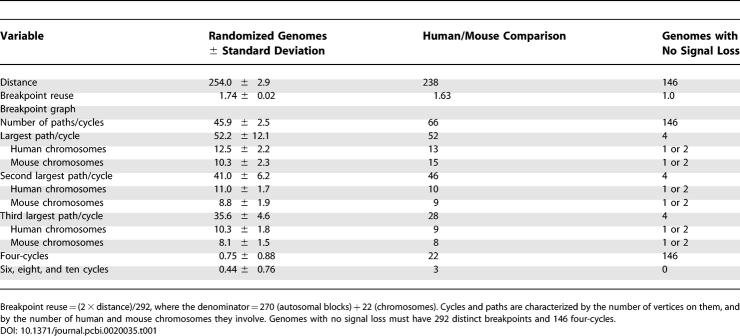
Human/Mouse Comparison Resembles Randomized Genome Comparison

The human/mouse comparison parallels the randomized genomes, and both deviate drastically from the hypothetical case of 270 blocks evolving without breakpoint reuse. There is an excess of 22 four-cycles and three other small cycles in the real data, largely due to reversals within concatenated blocks from a single chromosome in both human and mouse, largely dispersed in the randomized chromosomes. These 25 are what remains of the detailed evolutionary signal; they account for the small differences in distance, in breakpoint reuse, and in the total number of cycles. The giant cycles celebrated in Pevzner and Tesler's paper [6] and Tesler and colleagues' paper [3] have almost identical structure in the human/mouse and randomized comparisons.

Note that in contrast to the autosomes, the rearrangement analysis of the human and mouse X chromosomes involves only short cycles, a breakpoint reuse rate close to 1.0 and a clear evolutionary signal.

In conclusion, I take issue neither with Pevzner and Tesler's ingenious method for constructing synteny blocks nor with the notion that genomes are spatially heterogeneous in their susceptibility to rearrangement; many types of genomic regions, as reviewed in a previously published paper [5], have documented elevated rates of rearrangement. Nevertheless, a high reuse rate in the output of rearrangement algorithms, which simply indicates loss of signal, is not good evidence for fragile regions. The output of comparisons of randomized genomes has the same characteristics—namely, similar rearrangement distance, similar cycle/path sizes, similar number of chromosomes touched by each large cycle, similar reuse rates, and similar estimates [8] of the number of translocations and reversals.
